# Ascite fébrile chez la femme, ne pas méconnaitre une tumeur de Krukenberg

**DOI:** 10.11604/pamj.2015.21.269.7108

**Published:** 2015-08-11

**Authors:** Adama Berthé, Madoky Magatte Diop, Linda Bentefouet, Papa Abdoulaye Ba, Fulgence Abdou Faye, Papa Souleymane Touré, Marietou Thiam, Lamine Gueye, Bernard Marcel Diop, Mamadou Mourtalla Ka

**Affiliations:** 1 Université de Thiès - UFR des Sciences de la Santé Ex l0 ^ème^ Thiès, Sénégal; 2 Service de Médecine Interne Centre Hospitalier Régional de Thiès, 1 Avenue Malick SY Prolongée, Thiès Sénégal; 3 Service de Chirurgie Générale Centre Hospitalier Régional de Thiès, 1 Avenue Malick SY Prolongée, Thiès,Sénégal; 4 Service de Gynécologie-Obstétrique Centre Hospitalier Régional de Thiès, 1 Avenue Malick SY Prolongée, Thiès, Sénégal

**Keywords:** Métastases ovariennes, tumeur de Krukenberg, pronostic sombre, ovarian metastases, Krukenberg tumor, prognosis

## Abstract

Les tumeurs de Krukenberg (TK) se définissent comme des métastases ovariennes d'un cancer, le plus souvent digestif. Elles représentent 5 à 15% des tumeurs malignes ovariennes. Notre objectif était de décrire les caractéristiques épidémiologiques, diagnostiques, thérapeutiques et évolutives. Nous rapportons deux observations de tumeur de Krukenberg découvertes à l'occasion de l'exploration d'une ascite fébrile.Il s'agit de deux patientes multipares âgées respectivement de 32 ans et 50 ans. Les signes d'appel étaient essentiellement digestifs. La découverte de ces métastases ovariennes était survenue à distance des foyers primitifs. L'atteinte des ovaires était bilatérale dans le premier cas et unilatérale droite dans le second cas. Le diagnostic est apporté par la tomodensitométrie abdominopelvienne dans les deux cas. La fibroscopie œsogastroduodénale avait permis de retrouver le foyer primitif respectivement sous forme d'un processus bourgeonnant et d'un ulcère en position antrale avec des stigmates d'hémorragies. L'examen anatomopathologique des biopsies réalisées mettait en évidence un adénocarcinome tubuleux moyennement différencié de l'estomac avec composante mucineuse dans la première observation et un adénocarcinome de type intestinal moyennement différencié dans la seconde. Le traitement chirurgical confirme le diagnostic histologique. Dans notre série, le traitement n'a pu être que symptomatique en raison de l'existence constante d'une carcinose péritonéale et de l'altération profonde de l’état général. Les deux patientes ont été confiées à l'institut de cancérologie pour une chimiothérapie palliative. La première est décédée 1 mois après. La tumeur de Krukenberg est une maladie rare. Le diagnostic est facilité par la radiologie et confirmé par l'histologie. Son pronostic demeure encore très sombre. Le seul espoir réside dans les mesures préventives.

## Introduction

Les tumeurs de Krukenberg (TK) se définissent comme des métastases ovariennes d'un cancer, le plus souvent digestif. Elles représentent 5 à 15% des tumeurs malignes ovariennes secondaire [1]. Cependant, des formes primitives ont été décrites [2]. Le manque de spécificité des signes d'appel des TK, explique qu'elles sont habituellement diagnostiquées à un stade tardif. Ainsi, sont rapportés, pour la première fois au Sénégal, à notre connaissance, deux cas découverts à l'occasion d'une exploration d'une ascite fébrile. Le but de cette étude est de présenter les caractéristiques épidémiologiques, diagnostiques, thérapeutiques et évolutives de cette tumeur maligne secondaire de l'ovaire

## Patient et observation

**Figure 1:**
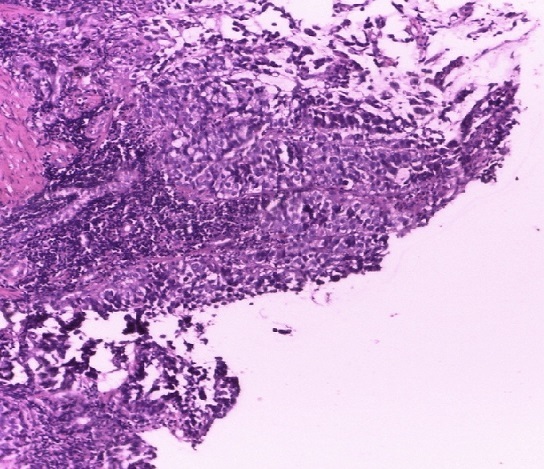
Adénocarcinome gastrique (grossissement x 10; coloration HE) Commentaire 01: il s'agit d'une prolifération tumorale maligne de cellules glandulaires atypiques organisées en massif et en travées et infiltrant un stroma fibreux

**Figure 2:**
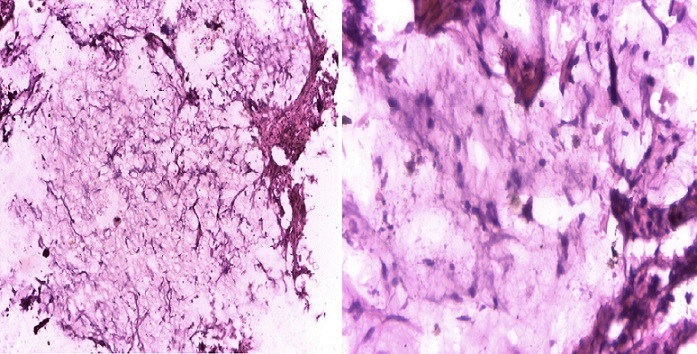
Adénocarcinome gastrique avec composante mixoide (grossissement x 10; coloration HE) Commentaire 02: sur ce champ, on observe une composante mucineuse très importante en mauve claire, parcourue de cellules tumorales atypiques (mauve foncé)

**Observation 1:** Mme M. A. T., 32 ans, 6 gestes et 6 pares, sans antécédents pathologiques connus, est adressée en médecine interne pour une exploration d'une ascite fébrile, associée à une altération de l’état général le 03 janvier 2015. L'anamnèse, révèle que la patiente présentait depuis quelques semaines une douleur épigastrique atroce, à type de brûlure, calmée par les vomissements postprandiaux précoces. A cela, s'ajoutaient une douleur pelvienne et une constipation opiniâtre sans notion de métrorragie. La douleur et cette constipation étaient antérieures à la douleur gastrique de quelques mois. L'examen clinique à l'admission retrouvait: un état général altéré, un pli cutané persistant de déshydratation, une ascite de moyenne abondance, sans splénomégalie ni circulation veineuse collatérale. Des nodules profonds sus-ombilicales et une sensibilité épigastrique étaient notés sans masse palpable. La fibroscopie œso-gastro-duodénale faisait observer une incontinence cardiale et un processus bourgeonnant de la portion inferieure s’étendant à tout l'antre englobant et sténosant le pylore dont la base était indurée. L'examen anatomo-pathologique des biopsies gastriques concluait à un adénocarcinome tubuleux moyennement différencié de l'estomac (Figure 1) avec composante mucineuse (Figure 2). La tomodensitométrie abdominale montrait: un épaississement circonférentiel, régulier de la paroi de la région antro-pylorique, un nodule tissulaire bilatéral des fosses ovariennes de 19 mm à droite et 22 mm à gauche, une densification de la graisse mésentérique et dans une moindre mesure du grand épiploon, une lésion focale hépatique du segment VI de taille centimétrique d'aspect kystique. Le tout évoquait un syndrome de Krukenberg associé à une tumeur infiltrante antro-pylorique avec carcinose péritonéale. Son tableau clinique rendait une chirurgie palliative impossible. Le délai diagnostic est estimé à 7 mois (délai entre apparition des premiers symptômes et son admission à l'hôpital). La patiente fut confiée à un oncologue pour une chimiothérapie palliative. Elle est décédée 1 mois après.

**Figure 3:**
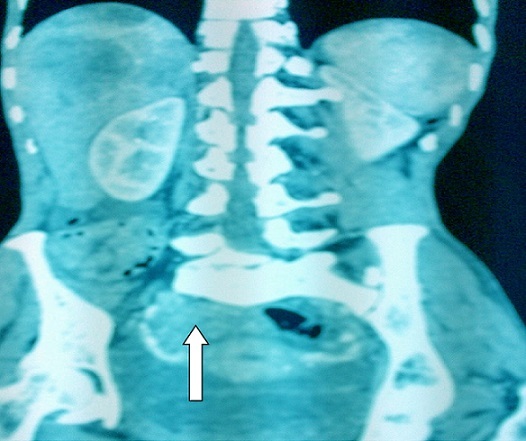
Image scannographique en coupe longitudinale montrant une masse tumorale ovarienne droite

**Figure 4:**
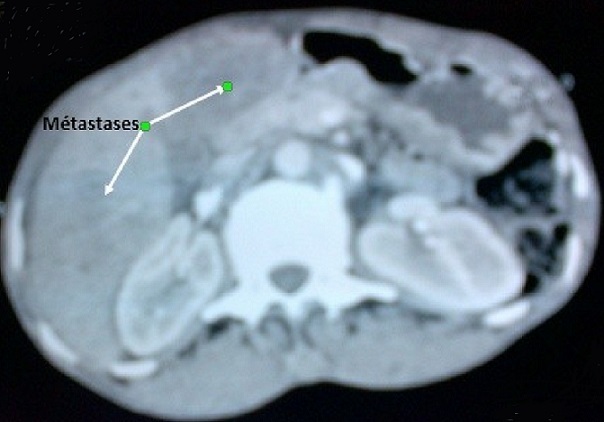
Image scannographique en coupe transversale montrant des métastases hépatiques

**Observation 2: p**atiente âgée de 50 ans, multigestes multipares, admise en médecine interne le 24 décembre 2014 pour une hématémèse de moyenne abondance, une douleur épigastrique d'allure ulcéreuse et une distension abdominale sans métrorragie. L'examen de la patiente à l'admission faisait observer un en mauvais état général, une pâleur des muqueuses, une ascite de moyenne abondance sans circulation veineuse collatérale ni splénomégalie. La fibroscopie œsogastroduodénale mettait en évidence un ulcère antral avec des stigmates d'hémorragies. Des biopsies systématiques furent réalisées. L'examen anatomopathologique de la biopsie antrale concluait à un adénocarcinome de type intestinal modérément différencié. Le délai diagnostic était d'un an. La tomodensitométrie abdominale révélait une masse tumorale ovarienne droite (Figure 3) avec des localisations hépatiques secondaires (Figure 4), des adénopathies profondes et une ascite libre de moyenne abondance. L'ensemble de ce tableau fit évoquer une tumeur de Krukenberg avec métastase hépatique et carcinose péritonéale. Devant l'altération de l’état général et la carcinose péritonéale, une chirurgie palliative n'a pas pu être réalisée. La patiente fut référée à l'institut de cancérologie pour une chimiothérapie palliative.

## Discussion

Le cancer de l'ovaire constitue le sixième cancer le plus fréquent chez la femme. Environ une femme sur 70 risque d'en être atteinte au cours de son existence [3]. Il s'agit du cancer le plus meurtrier en gynécologie avec un taux de létalité de plus de 60% [4]. Les métastases ovariennes encore appelées tumeurs de Krukenberg sont loin d’être rares. Elles représentent 5 à 15% des tumeurs malignes ovariennes [1]. La plupart des localisations primitives sont d'origine digestive notamment gastrique et colique [5, 6]. Rarement le point de départ est extra-digestif (sein, thyroïde) [7]. En Afrique subsaharienne, Attipo [8], en 1996, au Togo et Fallandry [9], en 1992, au Gabon ont rapporté respectivement 4 cas et 1 cas de TK. Nous rapportons deux cas supplémentaires de métastases ovariennes d'un adénocarcinome gastrique et intestinal révélé par une ascite fébrile. Cette affection survient avec une fréquence élevée chez la femme jeune en période d'activité génitale. L’âge habituel de survenu se situe entre 30 et 50 ans comme dans nos observations (32 ans et 50 ans). Toutefois, de rares cas, survenant chez une adolescente en 1998 [10] et une femme âgée en 2002 [11], sont décrits dans la littérature. La plupart des auteurs constatent que les femmes atteintes sont le plus souvent multipares [6, 12]. Ce constat est similaire pour nos deux observations. Du point de vue physiopathologique, la grande interrogation reste dominée par le mode de dissémination néoplasique qui peut exister entre un cancer du tractus digestif et la métastase ovarienne. Ainsi, plusieurs hypothèses ont été avancée parmi lesquelles, nous retenons la voie lymphatique rétrograde, le chimiotactisme, l'immuntactisme et l'hormonotactisme [13, 14]. Sur le plan clinique, les signes d'appel des tumeurs de Krukenberg sont frustres et non spécifiques. Ceci explique que le diagnostic est le plus souvent posé tardivement. Dans notre série les délais étaient respectivement de 7 mois à 1 an. Les symptômes digestifs vagues notamment la présence de l'ascite, les vomissements, la douleur épigastrique expliquent la fréquente orientation vers d'autres spécialités surtout en gastro-entérologie ou en médecine interne (comme c'est le cas pour nos patientes). Toutefois, la tumeur est de découverte fortuite dans la majorité des cas lors d'un bilan d'extension d'un cancer primitif le plus souvent digestif [11] ou en per opératoire [6, 15]. Chez nos patientes, les manifestations digestives ont masqué les symptômes en rapport avec la maladie ovarienne. La ponction exploratrice de l'ascite a ramené un liquide franchement hématique dans les 2 cas. L'examen cytologique et chimique du liquide retrouvait respectivement une hypercellularité lymphocytaire et une réaction de Rivalta positive. La recherche de cellules malignes n'a pas été effectuée chez nos patientes; elle aurait pu permettre la mise en évidence, au microscope optique, de cellules en bague à chaton qui secrètent de la mucine, caractéristique de la tumeur de Krukenberg [16]. Ainsi, ses résultats nous ont permis d’évoquer une carcinose péritonéale et de rechercher un foyer primitif.

En imagerie, les métastases ovariennes se présentent sous forme de masses majoritairement tissulaires, siège de quelques images kystiques intramurales bien limitées [17]. Toutefois, l’échographie pelvienne n’était pas contributive chez nos malades. En effet, en per-opératoire, l’échographie et le scanner n'objectivent que les tumeurs volumineuses [18]. Dans nos observations, le scanner a confirmé la nature ovarienne de la tumeur. L'endoscopie haute a permis de retrouver le foyer primitif respectivement sous forme d'une tumeur gastro-pylorique stenosante et d'un ulcère antral avec des stigmates d'hémorragies. La tomodensitométrie n'a pas une grande valeur diagnostique, elle montre les signes de malignité de la tumeur ovarienne, masse solido-kystique multilobulaire, mais ne permet en aucun cas de différencier une tumeur ovarienne primitive d'une tumeur secondaire [19]. Par contre, elle occupe une place primordiale dans le diagnostic de la tumeur primitive, et reste un élément essentiel dans le bilan d'extension. Choi [20], dans une étude comparative de l'aspect scannographique entre les métastases ovariennes provenant d'un primitif colique et gastrique, constate que les métastases ovariennes provenant de l'estomac apparaissent plus denses vu que leur composante tissulaire est plus importante. Au plan hormonal, le CA 125 est le marqueur le plus fréquemment utilisé par les auteurs dans les tumeurs de Krukenberg. En fait il est le marqueur le plus souvent élevé. Il peut jouer un rôle dans le dépistage précoce des métastases ovariennes, dans le suivi, et même le pronostic. Les auteurs ont constaté que la survie est inversement proportionnelle avec le taux du CA125 [16]. Au plan anatomopathologique, l'atteinte des ovaires est le plus souvent bilatérale (80% dans la littérature). Cette caractéristique de bilatéralité des tumeurs de Krukenberg est un argument de plus de leur nature métastatique [16]. Rappelons que ces tumeurs ont un aspect macroscopique bénin. La surface externe est soit bosselée ou polylobée, soit parfaitement lisse reproduisant la forme générale de l'ovaire, sans adhérence aux organes de voisinage [21, 22]. Dans nos observations, nous avons constaté un cas de bilatéralité et un cas unilatéral droit. La localisation ovarienne droite isolée est estimée entre 9 et 28% [23]. Quant à l'atteinte gauche isolée, elle varie entre 40 et 50% des cas dans la littérature [23]. Pour les auteurs, même si un des ovaires apparaît sain macroscopiquement, la métastase est presque toujours retrouvée des deux cotés à l'histologie. L’étude histologique est la seule à pouvoir affirmer le diagnostic. Chez nos patientes, aucune n'a pu bénéficier de diagnostic histologique du fait de la carcinose péritonéale rendant inutile tout geste chirurgical.

L'immunohistochimie montre une positivité au CK 7. La biologie moléculaire est d´un apport fort intéressant et permet de différencier une origine primitive ou secondaire de la masse ovarienne en cas de doute diagnostic [24] et ce par la technique de recherche de perte chromosomique à l´aide de marqueurs microsatellites. La TK peut être associée à la grossesse. Cette association est rare [25, 26] et son diagnostic n'est pas toujours aisé. Les signes cliniques ne sont pas spécifiques. Il s'agit le plus souvent de symptômes gastro-intestinaux (nausée, vomissement, dyspepsie, constipation), qui sont souvent à tort rattachés à la grossesse. La persistance de ces symptômes au-delà du premier trimestre justifierait l'investigation, et il ne faut pas hésiter à redemander les explorations devant un examen complémentaire réalisé plusieurs semaines ou mois auparavant. Le traitement de la tumeur de Krukenberg est toujours chirurgical. Il est basé sur une exérèse la plus précoce possible de la tumeur primitive, suivie d'une hystérectomie totale avec ovariectomie bilatérale. Le traitement chirurgical confirme le diagnostic histologique et prévient les complications engendrées par l’évolution locale et régionale de la tumeur. De plus, il apporte un confort de survie relativement satisfaisant. Dans notre série, le traitement n'a pu être que symptomatique en raison de l'existence constante d'une carcinose péritonéale. Quant à la chimiothérapie et à la radiothérapie palliatives [6, 12], elles n'ont pas encore fait la preuve de leur efficacité. La chimiothérapie est prescrite en général en postopératoire, ou à titre exclusif lorsque les malades ne sont pas opérables. Pour nombreux auteurs c'est un excellent complément thérapeutique à la chirurgie, occupant une place importante dans l'arsenal thérapeutique. Elle doit être entamée le plus tôt possible après l'acte chirurgical [27]. Devant l'altération profonde de l’état général et la carcinose péritonéale, nos malades n'ont pas pu bénéficier d'une chirurgie palliative. L'une est décédée à 1 mois avant la chimiothérapie (observation 1) tandis que l'autre a débuté les séances.

## Conclusion

La tumeur de Krukenberg est une tumeur métastatique, mystérieuse et suscite encore de nombreuses interrogations. Le pronostic reste encore très sombre avec une moyenne de survie de 12 mois après le diagnostic. Le dépistage systématique doit passer nécessairement par un examen gynécologique complet devant toute symptomatologie digestive néoplasique.
